# Influence of COVID-19 Restrictions on Training and Physiological Characteristics in U23 Elite Cyclists

**DOI:** 10.3390/jfmk7010001

**Published:** 2021-12-21

**Authors:** Peter Leo, Iñigo Mujika, Justin Lawley

**Affiliations:** 1 Division of Performance Physiology & Prevention, Department of Sport Science, University of Innsbruck, 6020 Innsbruck, Austria; justin.lawley@uibk.ac.at; 2 Department of Physiology, Faculty of Medicine and Nursing, University of the Basque Country, 48940 Leioa, Spain; inigo.mujika@inigomujika.com; 3 Exercise Science Laboratory, School of Kinesiology, Faculty of Medicine, Universidad Finis Terrae, Santiago 8320000, Chile

**Keywords:** pandemic, athletes, endurance, detraining, cycling

## Abstract

PURPOSE: The COVID-19 pandemic and its associated mobility restrictions caused many athletes to adjust or reduce their usual training load. The aim of this study was to investigate how the COVID-19 restrictions affected training and performance physiology measures in U23 elite cyclists. METHODS: Twelve U23 elite cyclists (*n* = 12) participated in this study (mean ± SD: Age 21.2 ± 1.2 years; height 182.9 ± 4.7 cm; body mass 71.4 ± 6.5 kg). Training characteristics were assessed between 30 days pre, during, and post COVID-19 restrictions, respectively. The physiological assessment in the laboratory was 30 days pre and post COVID-19 restrictions and included maximum oxygen uptake (V̇O_2max_), peak power output for sprint (Sprint_Pmax_), and ramp incremental graded exercise (GXT_Pmax_), as well as power output at ventilatory threshold (VT) and respiratory compensation point (RCP). RESULTS: Training load characteristics before, during, and after the lockdown remained statistically unchanged (*p* > 0.05) despite large effects (>0.8) with mean reductions of 4.7 to 25.0% during COVID-19 restrictions. There were no significant differences in maximal and submaximal power outputs, as well as relative and absolute V̇O_2max_ between pre and post COVID-19 restrictions (*p* > 0.05) with small to moderate effects. DISCUSSION: These results indicate that COVID-19 restrictions did not negatively affect training characteristics and physiological performance measures in U23 elite cyclists for a period of <30 days. In contrast with recent reports on professional cyclists and other elite level athletes, these findings reveal that as long as athletes are able to maintain and/or slightly adapt their training routine, physiological performance variables remain stable.

## 1. Introduction

Endurance athletes are known for performing high training volumes throughout the year to maintain cardiovascular fitness and to attain peak performance for the competition period [1,2,3]. The COVID-19 pandemic has been reported to impact the athletes’ training, competition, and recovery routines [4,5,6,7,8,9]. Washif et al. [4] analyzed the training habits of 12,526 athletes of various performance levels (i.e., recreational to professional) from 142 countries and six continents during the COVID-19 confinement. Despite reductions in training session frequency, most athletes focused on maintenance of general endurance and strength rather than exploiting sports-specific training [4,10]. Muriel et al. [11] studied the training and physiological characteristics of 18 male professional cyclists during the 10 weeks prior to the COVID-19 confinement and during the seven-week confinement period: Total training volume significantly decreased by 33.9% during the lockdown. Weekly volumes (hours per week) by standardized training zones declined between 25.8% and 52.2%. There were also large reductions in the best 5-min and 20-min power outputs with declines between 1% and 19% in all the cyclists.

There is increasing evidence that insufficient training stimuli impair major physiological determinants for endurance performance in both the general population [12] and highly trained athletes [13]. Mujika and Padilla [13] reported that training cessation over four weeks causes a rapid decline of 6% to 20% in 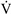
O_2max_, 10% to 14% in maximal ventilatory volume, 5% to 12% in blood volume and plasma volume, as well as inducing reductions in cardiac dimensions (e.g., left ventricular posterior wall thickness) by 25%.

As the current pandemic situation presents a new challenge for athletes and practitioners, there is limited evidence about the acute effects of a lockdown and mobility restriction period on road cyclists’ training characteristics and physiological determinants of performance. Therefore, the aim of the present study was to investigate how COVID-19 restrictions affected pre and post Sprint_Pmax_, GXT_Pmax_, 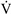
O_2max_, and submaximal thresholds in U23 elite cyclists. Additionally, training characteristics were compared between 30 days pre, during, and post COVID-19 restrictions. We hypothesized that COVID-19 restrictions would negatively affect physiological performance variables and training characteristics in U23 elite cyclists.

## 2. Materials and Methods

### 2.1. Participants

Twelve U23 elite cyclists of a Union Cycliste Internationale (UCI) licensed continental team participated in this study (mean ± SD: Age 21.2 ± 1.2 years; height 182.9 ± 4.7 cm; body mass 71.4 ± 6.5 kg). All methods were approved by the ethics committee under the conditions of the Declaration of Helsinki. All subjects voluntarily participated in the study and provided informed written consent.

### 2.2. COVID-19 Restrictions

The whole intervention involved 90 days, which were equally divided into 30 days pre, during, and post COVID-19 restrictions. Participants were living either in Austria (*n* = 6), Germany (*n* = 4), or Italy (*n* = 2). COVID-19 restriction guidelines were followed according to each country’s own regulations over 30 consecutive days and included mobility restrictions (*n* = 10) and home confinement (*n* = 2).

### 2.3. Training Characteristics

The accumulated training hours, distance covered, and training frequency per week were recorded for the respective periods, mentioned above. All athletes uploaded their training data to an online training platform (Trainingpeaks, Trainingpeaks LLC, Winchester Cir, MA, USA) [14]. Weekly training hours, distance covered, and training frequency were collected and further processed, analyzed, and checked for data spikes in Microsoft Excel (Excel, Microsoft Corporation, Redmond, WA, USA). Intensity ratios, including distance per hour (km·hour^−1^) and distance per session frequency (km·session^−1^), were calculated.

### 2.4. Physiological Performance Measures

Physiological performance measures were assessed 30 days before and after the COVID-19 restrictions. All laboratory tests were completed on an electromagnetically braked stationary trainer (Cyclus2, RBM electronic-automation GmbG, Leibzig, Deutschland) with the participants’ own road bikes (Alto Prestige, KTM Fahrrad GmbH, Mattighofen, Austria). The testing protocol involved a standardized warm-up of 5 min at 100 W, followed by a 15-s sprint test and a laboratory incremental graded exercise test (GXT). The cycling cadence was reduced to 20–30 revolutions per minute (rpm), and the sprint started after a 5-s countdown. After a 10-min recovery phase at 50 W, the GXT was completed. The initial workload for the GXT was set at 150 W and was increased by 20 W per min until volitional exhaustion. The measurements included absolute and relative Sprint_Pmax_ for the 15 s sprint test as well as GXT_Pmax_. 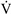
O_2max_ was defined as the highest 30 s average during the GXT [15]. VT and RCP were analyzed from the GXT, according to Beaver et al. [16]. VT was defined as the point where the ventilation rate (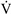
E) increased compared to 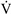
O2 (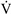
E/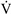
O2). RCP was defined as the onset of hyperventilation during the GXT, with an increase in 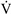
E compared to the volume of carbon dioxide (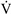
CO2) release, known as the 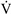
E/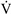
CO2 ratio. Open circuit spiro-ergometry (ZAN600, nSpire Health GmbH, Germany) with a flow sensor (FlowSensor, Type II, nSpire Health GmbG, Oberthulba, Germany) was used to record oxygen uptake (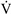
O2) and carbon dioxide (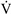
CO2) release. Continuous recordings of heart rate (HR) were performed via short range telemetry with a 1 Hz sampling rate (V800, Polar Electro Oy, Kempele, Finland).

### 2.5. Statistical Analysis

All recorded data were initially checked for violations to normality with a Shapiro-Wilk test. Paired samples *t*-tests with Cohen’s d effect size were performed between all physiological performance characteristics. Cohen’s d was set at 0.2 for small, 0.5 for moderate, and 0.8 for a large effect [17]. A one-way repeated measure analysis of variance (ANOVA) was conducted to compare training characteristics for the 30 days pre, during, and post COVID-19 restrictions. Partial eta square (partial η^2^) effect size was set at 0.01 (small), 0.06 (moderate) and 0.14 for a large effect. All statistical analyses (JASP 0.15, JASP Team, Amsterdam, The Netherlands) and graphical illustrations (GraphPad Prism 8, GraphPad Software, San Diego, CA, USA) were conducted with commercially available software packages.

## 3. Results

The participants’ anthropometric and physiological characteristics are presented in Table 1 (mean ± SD).

### Training Characteristics

**Figure 1:**
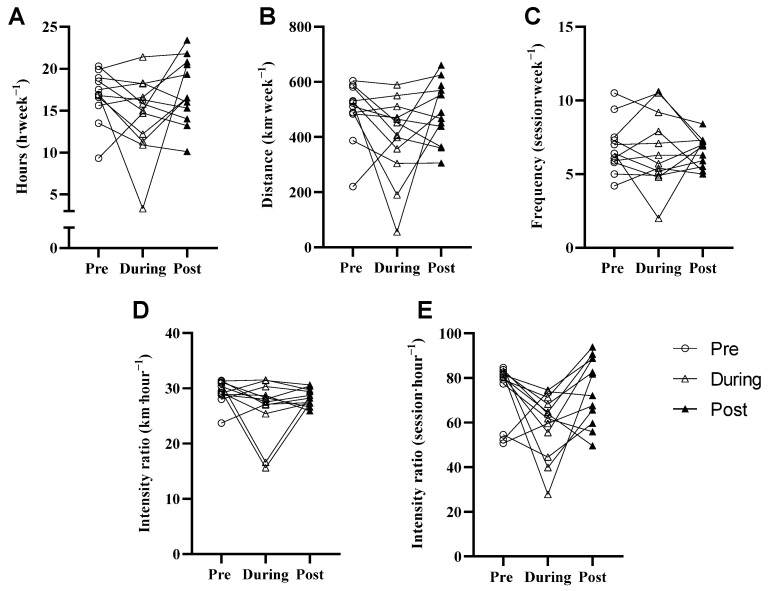
Non-significant differences in training hours (**A**), distance (**B**), frequency (**C**) and intensity ratios (**D**,**E**) between 30 days pre, during, and post COVID-19 restrictions. Physiological Performance Measures.

No statistical difference was found in training volume, including completed hours (partial η^2^: 0.175), covered distance (partial η^2^: 0.227), and session frequency (partial η^2^: 0.030) in the 30 days pre, during, and post COVID-19 restrictions (*p* > 0.05). Intensity ratios km·hour^−1^ (partial η^2^: 0.182) and km·session^−1^ (partial η^2^: 0.187) also remained unchanged in the 30 days pre, during, and post COVID-19 restrictions. (*p* > 0.05)—see Figure 1.

**Figure 2:**
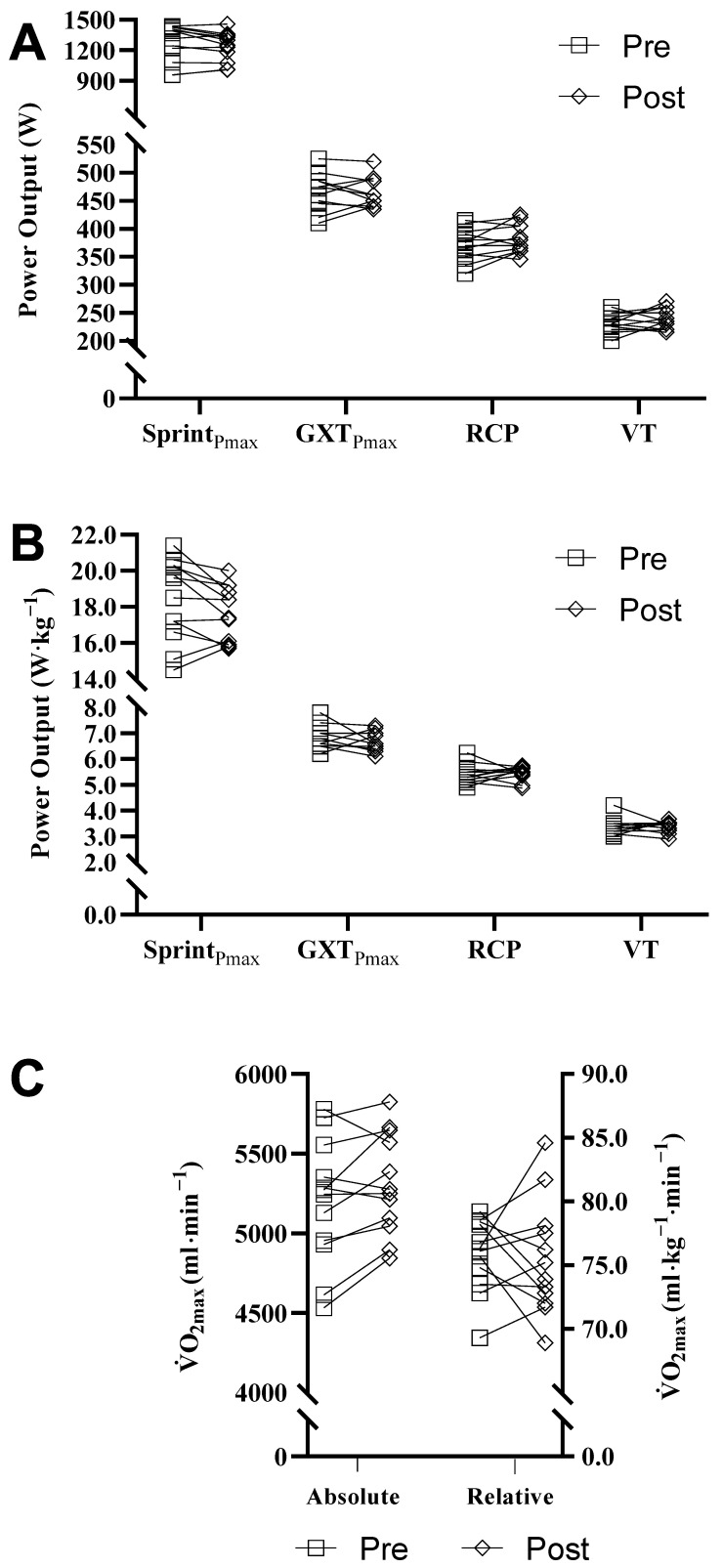
Non-significant differences in physiological performance measures between pre and post COVID-19 restrictions; Absolute (**A**) and relative (**B**) peak power output in Sprint_Pmax_—peak power in the laboratory sprint test; GXT_Pmax_—laboratory graded incremental exercise test; RCP—respiratory compensation point; VT—ventilatory threshold; 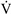
O_2max_—maximum oxygen uptake (**C**).

No statistical differences were found in absolute and relative Sprint_Pmax_ (effect size (ES): 0.451, 0.596, respectively), GXT_Pmax_ (ES: 0.056, 0.198, respectively), power at VT (ES: −0.302, −0.083, respectively) and RCP (ES: −0.522, −0.096, respectively), HRmax (ES: 0.123) as well as absolute and relative 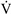
O_2max_ (ES: −0.622, −0.071, respectively), between pre and post COVID-19 restrictions—see Figure 2.

## 4. Discussion

In contrast to our own initial hypothesis, the present study showed no differences in training characteristics and physiological performance determinants in U23 elite cyclists.

To the authors’ knowledge, this is the first study to investigate the effects of COVID-19 restrictions on a U23 elite cycling population. While there has been research conducted on professional cyclists by Muriel et al. [18], their findings were not in line with those of the present study. Muriel et al.’s study [18] reported a significant decline in training characteristics and physiological performance variables during the seven weeks of the COVID-19 confinement in a Spanish professional cycling team. In contrast, the findings of the present paper show that the four-week COVID-19 restrictions did not have a negative effect on training characteristics and physiological performance variables, including 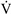
O_2max_ and power output at submaximal thresholds. These conflicting findings might lead to the assumption that the COVID-19 restriction policies were different across European countries [19]. The study’s participants living in northern European countries could continue their normal training habits (mobility restrictions), while participants living in southern European countries were restricted to indoor training (home confinement). These differences underpin why the subjects in Muriel et al.’s study [18] indicated reduced training volume because they were forced to train indoors (home confinement). Washif et al. [4] undertook a worldwide online survey with 12,526 athletes, including male and female professional and amateur athletes. While most athletes were training more individually to maintain general fitness and health, most athletes reported reduced motivation due to a lack of competitive events. Although professional athletes were coping better with the COVID-19 confinement than amateur athletes, all populations reported reductions in training volume, intensity, and frequency. While these data are based on qualitative assessments (survey), our empirical (quantitative) assessment does not confirm this trend. Despite average reductions in training hours (−15.1% to −18.6%), distance (−23.7% to −25.0%), session frequency (−4.7% to −6.3%), and intensity ratios (−7.2% to −19.1%) compared to pre and post COVID-19 restrictions, the inter-individual variations were too large to result in a significant statistical change. In addition, the small sample size associated with a reduced statistical power increases the likelihood of a false “negative” null hypothesis, known as a Type I error [20]. To better interpret inter-individual differences, effect sizes are provided. “Large” effects (partial η^2^ > 0.14) were found in training characteristics between pre, during, and post COVID-19 restriction periods. The reason for those discrepancies could be due to different COVID-19 restrictions between European countries [19]. Two participants of the present study who experienced home confinement automatically indicated reduced training volume, while the other 10 participants were able to continue their normal training habits. This might explain why collectively the statistical differences remained non-significant. Reductions in training volume during the COVID-19 restrictions were also observed in elite swimmers, which were linked to decreased vagal activity [10].

This study also investigated the change in physiological performance determinants pre and post COVID-19 restrictions. Relative GXT_Pmax_ and Sprint_Pmax_ decreased on average by 0.2 ± 1.3% to 4.3 ± 0.5%, respectively, which was mainly due to a 1.7 ± 0.1% increase in body mass. This change in body mass between pre and post COVID-19 restrictions affected average 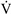
O_2max_, and power output at RCP and VT when normalized to body mass in the range of −0.5% to −0.9%. Interestingly, average submaximal power outputs at RCP and VT slightly improved, despite small reductions in 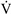
O_2max_, GXT_Pmax_, and Sprint_Pmax_. However, statistical significance was not achieved between pre and post COVID-19 restrictions in any of the physiological performance parameters assessed, showing small (<0.2) to moderate (0.6) effects.

Collectively, our data do not support our initial hypothesis that COVID-19 restrictions would negatively affect training and physiological performance characteristics. Despite declining trends in training characteristics when compared to pre and post COVID-19 restrictions, inter-individual variations were too large to reach statistical significance.

## 5. Limitations

This study is not without its limitations. The findings need to be interpreted and discussed cautiously due to a small sample size and country-specific COVID-19 restrictions.

First, the COVID-19 restrictions were country specific and differently affected the training habits of our participants. For this reason, the most restricted athletes indicated the biggest decline in training characteristics but not in performance. Secondly undertaking research in highly trained athletes is challenging and rare due to team commitments, busy racing schedules and logistical challenges. Additionally, using standardized laboratory testing to inform the present research design and experiment was difficult due to the COVID-19 situation. Although a bigger sample size of 20–30 athletes would have been beneficial to improve the statistical power, we strongly believe that reporting physiological data in this subgroup of highly trained athletes represents an important contribution to the existing literature. Moreover, qualitative assessments (i.e., survey) were not undertaken in this study, which could have been valuable in explaining inter-individual differences for the training process. In addition, measures of psychological state and well-being would have also been relevant to monitor the participants’ coping strategies during COVID-19 restriction periods.

## 6. Conclusions

The present study demonstrates no statistically significant changes in training characteristics and physiological performance variables during COVID-19 restrictions. These findings need to be interpreted cautiously as country specific COVID-19 regulations and a small sample size with limited statistical power clearly influenced the study’s outcome. However, the study’s data reveal that if athletes maintain around 75% of their training volume, physiological performance measures remain stable at least for periods of up to 30 days.

## Figures and Tables

**Table 1:** Non-significant differences in anthropometrical and physiological measures between pre and post COVID-19 restrictions; BMI—body mass index; HRmax—maximum heart rate; GXT_Pmax_—peak power in the laboratory graded incremental exercise test; 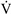
O_2max_—maximum oxygen uptake.

	Body Mass (kg)	BMI (kg·m^−2^)	HR_max_ (bpm)	GXT_Pmax_ (W)	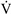 O_2max_ (mL·kg^−1^·min^−1^)
PRE	68.8 ± 3.8	20.7 ± 0.7	194 ± 7	471 ± 36	75.4 ± 4.4
POST	70.1 ± 4.4	21.1 ± 0.9	193 ± 5	470 ± 30	75.8 ± 2.8

## Data Availability

The datasets generated during and/or analysed during the current study are available from the corresponding author on reasonable request.
